# The Relationship between F_2_-Isoprostanes Plasma Levels and Depression Symptoms in Healthy Older Adults

**DOI:** 10.3390/antiox11050822

**Published:** 2022-04-22

**Authors:** Karen Savage, Lee Gogarty, Ana Lea, Saurenne Deleuil, Karen Nolidin, Kevin Croft, Con Stough

**Affiliations:** 1 Centre for Human Psychopharmacology, School of Health Sciences, Swinburne University of Technology, Melbourne 3122, Australia; brainstormpsych@gmail.com (L.G.); analea@swin.edu.au (A.L.); saurenne@gmail.com (S.D.); knolidin@swin.edu.au (K.N.); cstough@swin.edu.au (C.S.); 2 Professorial Unit, The Melbourne Clinic, Department of Psychiatry, University of Melbourne, Melbourne 3121, Australia; 3 School of Biomedical Science, The University of Western Australia, Crawley 6009, Australia; kevin.croft@uwa.edu.au

**Keywords:** mood, depression, F_2_-Isoprostanes, ageing/aging, oxidative stress, sex, sex differences, body mass index

## Abstract

The increasing proportion of older citizens in our society reflects a need to better understand age-related biological underpinnings of mood, as depression in older age may be under-diagnosed. Pre-clinical and human studies evidence a relationship between oxidative stress (OS) biomarkers in depression symptoms, and an influence of biological factors such as Body Mass Index (BMI), but focus has been clinical or younger samples, and less is known about patterns in healthy older adults. We investigated these associations with data derived from the Australian Research Council Longevity Study (ARCLI; ANZCTR12611000487910), in 568 healthy adults aged 60–75 years using F_2_-Isoprostanes plasma levels, and controlling for demographic factors, in assessing mood via the Beck Depression Inventory-II, Chalder Fatigue Scale, and General Health Questionnaire 12. Elevated F_2_-Isoprostanes contributed to depressed mood on the BDI-II and reduced general health on the GHQ-12. BMI was positively associated with Chalder Fatigue scores, yet better ratings on the GHQ-12. Females had significantly higher F_2_-Isoprostanes than males. The results suggest that in otherwise healthy older adults, mood and mental health are reduced with increases in oxidative stress markers, exhibiting similar patterns observed in clinical groups. Sex as a factor should be considered when assessing OS levels in systemic pathologies. BMI as a modifiable risk factor for maintenance of mental health, and OS modification through nutrient supplementation, are discussed. The findings contribute to understanding oxidative stress marker patterns in healthy older adults and their potential role in mood symptoms and mental health.

## 1. Introduction

Globally, there is an unprecedented increase in the proportion of older citizens [1], and so the maintenance of health and wellbeing in older age is crucial to an optimal quality of life. Epidemiological studies suggest prevalence of mood and anxiety symptoms in 20% of adults aged above 60 [2,3,4]. The data also indicate that depressed mood may be more common than clinical depressive disorders in older populations [5,6], where symptoms are frequently attributed to other conditions such as medication or cognitive impairment [6,7,8,9] and which, taken together, suggest that depressed mood may be higher than evidenced [4,5,9,10], and therefore under-diagnosed, and under-treated. Older adults experiencing clinical depression are also at greater risk of dementias and chronic diseases, as well as higher mortality rates, including increased prevalence of suicidality than younger populations [11,12,13]. Moreover, the functional disablement occurring with late-life depressive disorders is more severe [8,9].

Mental health in older adults is influenced by declining physical health and cognition, and in many, the development of neurological disorders. Additionally, reduced activities and social support and increased exposure to stressful life events are strong contributors to poor mental health [3,6,7,8]. Despite these age-related factors, older adults are evidenced to rate their mental health as better than younger cohorts feasibly due in part to the benefits of life experience and reported resilience [14,15], as demonstrated in recent COVID-19 pandemic research [16]. The influence of physical health on mental health is therefore of great interest in aging research.

The mechanisms of aging may be defined as a progressive reduction in optimal homeostatic biological processes as a product of endogenous and environmental influences, that can be quantified via neurological, cognitive, and psychological ‘symptoms’ [17,18,19]. Investigating these patterns of functional decline in biological processes that occur with aging, and the subsequent impact on psychological health, will aid in improved identification and management of depression and anxiety symptoms, and treatment of clinical depression in older adults.

### 1.1. Oxidative Stress, Aging and Mood

Depressed mood, obesity, and cardiovascular health during aging have shared pathophysiological mechanisms including oxidative stress, hypothalamic–pituitary–adrenal axis chronic stress responses, neuroinflammation, and endothelial dysfunction [20,21,22,23,24,25,26,27,28,29,30]. Free-radical theory is a prominent model of biological aging along with genetic, proteomic and mitochondrial aetiologies [31,32], where elevations in oxidative stress (OS) are observed with increased age [33,34], producing a plethora of research into oxidative stress mechanisms [35,36]. Whilst antioxidants and free radicals consisting of reactive oxygen species (ROS) and reactive nitrogen species (RNS) are part of normal homeostatic function, OS occurs with imbalance between the two [22,28] and a key element of successful aging may be sustained ability to keep ROS/RNS production under control and maintain antioxidant capacity [34]. Following identification of F_2_-Isoprostanes (8-iso-PGF2-α) [37], a series of prostaglandin-like compounds, many studies have substantiated modulations across clinical disorders, establishing its use as a reliable systemic OS biomarker [38].

Elevated F_2_-Isoprostanes are observed in clinical conditions such as diabetes, cancer, cardiovascular disease and metabolic syndrome [39,40,41,42,43], for which age is also an independent risk factor. OS elevations are observed in psychiatric disorders, including depression [43,44,45], anxiety [46,47], and bipolar disorder [48], and the exact mechanisms of which remain to be elucidated. The research is limited in non-clinical samples and our existing depressed mood-OS understandings are derived from studies in younger adults, and only a handful of studies within older adults who may be more vulnerable to chronic OS elevations [49,50,51,52,53]. The presence of elevated OS in psychiatric disorders suggests that these conditions (where negative mood states prominently feature) are linked to systemic stressors in the maintenance of biological health, the relationship of which should be studied across all stages of the lifespan to better understand the mechanisms outside of clinical groups.

Between-study heterogeneity can greatly influence biological psychology studies; where variability in OS assay method is a confound that is prominent in OS research thus far, and the literature also exhibits diversity in methods of quantifying ‘depression’ [21,27,43,44,45,53]. Therefore, assessing the relationship between a gold-standard OS marker and levels of depressed mood and general mental health in older samples without chronic conditions is an important step in understanding OS influence on mood in biological aging.

### 1.2. Obesity, BMI, and Mood

At least one-third of the population globally are defined as overweight or obese, and two-thirds of Australians aged above 60 fall into this category [54,55,56]. Higher weight/body adiposity is observed in older compared with younger groups [57,58]. Obesity is observed with several comorbid conditions, such as haemodynamic, renal, and neurohormonal conditions, and with obesity-induced hypertension, where 70% of primary care setting patients with overweight or obese BMI levels exhibit hypertension [59,60,61,62]. Notably, higher BMI levels frequently co-occur with raised OS markers [63,64], including F_2_-Isoprostanes [65] and both OS and obesity are evidenced as predictors of cardiovascular disease in longitudinal studies, as well as cognitive decline, dementias, and impaired quality of life on older age [66,67,68]. The maintenance of a healthy weight in mid-to-late adulthood is a crucial modifiable risk factor for many of the aforementioned physical conditions [69].

Depression and obesity have a reciprocal relationship [70,71]; overweight and obese weight status is predictive of depression at 16 years follow-up in one study [72]. Both are risk factors for cardiovascular disease, in turn exerting additional pathologies over time. However, limited studies have examined weight and negative mood relationships in older groups beyond clinical samples, but the literature does suggest a U-shaped trend; positive associations in overweight/obese samples, and negative in cases of underweight BMI, where reduced nutrition, physical inactivity, and illness are factors [73,74,75,76]. A corollary to research into BMI and mood are findings from intervention studies of improved mood with physical activity, where it has been associated with lower incidence of reported depression symptoms, and Cochrane reviews have found modest evidence of physical exercise efficacy in the treatment of clinical depression [77,78,79].

### 1.3. Sex as a Biological Health Factor

Several studies support a higher prevalence of depression in women across the life span [3,80], which in turn promotes obesity risk (18%) [81]. A lower incidence of cardiovascular disease in females appears to equalize post-menopause where reduced oestrogens may precipitate increased visceral adipose tissue and hypertension [82,83].

There are limited and mixed findings related to OS patterns and sex differences in non-clinical adult samples, but again suggest a protective effect of estradiol pre-menopause and reduced benefit following menopausal cessation of the menstrual cycle. In women of reproductive age, F_2_-Isoprostanes fluctuate up to 66% through the menstrual cycle [84], supporting OS-endogenous hormonal activity linkage. Incorporating mood, the evidence is limited in healthy older adults: Milaneschi et al. (*m* = 74.6 years) [51] found higher OS levels and a greater incidence of depression in females, but a positive relationship between OS and depression was observed in males only. In clinical younger samples, Major Depressive Disorder (MDD) and bipolar disorder (BD) females showed higher OS levels compared with males [85], yet middle-aged samples, BD males appeared to have elevated OS compared with females [86].

Further exploration of OS, sex, and mood in older adults is essential to better understand how they may uniquely and together contribute to mental health. Identifying which factors in an individual represents risk can facilitate better management of health and mood in later life. What appears lacking in the literature is an examination of markers of general health and normative mood levels in tightly-controlled cohorts of older adults to better gauge this relationship in ‘healthy aging’. To this end, we investigated whether F_2_-Isoprostane levels, a validated biomarker of oxidative stress, was associated with negative mood in a healthy older adult sample, without diagnosed neurological, psychiatric, or chronic illness, meeting strict control criteria for health and lifestyle factors. We also assessed age (60–75 years), BMI, and biological sex as factors. It was predicted there would be sex differences in OS levels, and that higher F_2_-Isoprostane concentrations, and higher BMI, would also exhibit increased depressive symptom scores (therefore lowered mood) in this non-clinical older adult sample.

## 2. Materials and Methods

### 2.1. Design

Data were utilised from the baseline visit of the Australian Research Council Longevity Intervention study (ARCLI) [87]. ARCLI is a 52-week intervention study, in a randomised, placebo-controlled three-armed design examining multiple biochemical, cognitive, and psychological measures of aging and health in healthy older volunteers. This trial was registered on the Australian and New Zealand Clinical Trials Registry (ANZCTR12611000487910). The trial received Ethics approval from Swinburne University Human Research Ethics Committee and was conducted according to the Declaration of Helsinki.

### 2.2. Participants

The sample comprised 568 healthy older participants (male *n* = 216) aged 60–75 years from the ARCLI study cohort who had provided F_2_-Isoprostane data from blood samples during the baseline visit. Participants were in good general health without psychiatric, gastrointestinal or endocrine disorders, and without chronic illness in the previous 6 months (e.g., depressive episodes, influenza, and hepatitis A). Cardiovascular status was assessed more comprehensively; refer to Section 2.4.3 and Section 2.5.1 below.

### 2.3. Participant Recruitment

Participants were recruited by social media advertising, community groups, via an existing database of consenting participants, and word-of-mouth. Participants were reimbursed for their time and for out-of-pocket expenses during the trial.

### 2.4. Screening Measures

#### 2.4.1. Assessment of Cognitive Function

The Mini Mental Status Examination (MMSE) [88] with a minimum required score of 24 or above for enrolment, was administered at screening to detect impairments to cognition across six cognitive domains measured include orientation, attention, immediate recall, short-term recall, language, and ability to follow simple verbal and written instructions.

#### 2.4.2. Assessment of Depression Level

To ensure no prevalence of severe or clinical depressive symptoms, the Geriatric Depression Scale 15-item (GDS-15) [89], was administered with a score of 10 or less for enrolment. The GDS has sound psychometric properties in ascertaining above-normal levels [90]. Scores suggest the following depressive symptom severity; normal (0–9); mild (10–19); and severe (20–30).

#### 2.4.3. Assessment of Medical Eligibility

Comprehensive medical status including cardiovascular, neurological, and medication use (past and current) were assessed at screening and collected at the baseline visit. An established medical committee (research nurse, general practitioner, and ARCLI chief investigator) assessed all medical conditions prior and emergent during enrolment, in particular the presence and type of cardiovascular disease (CVD) which needed to be medically-managed and within ‘normal’ ranges (refer to [87]). Participants were generally unmedicated; with certain exceptions (respiratory; *pro re nata* anti-inflammatories), and selected supplements (minerals, vitamin D, omega 3). Alcohol use was screened, inclusive of women reporting <14 standard drinks (10 g alcohol) and men with <28 standard drinks weekly [91].

### 2.5. Health and Lifestyle Factors

#### 2.5.1. Cardiovascular Status

Cardiovascular conditions were allowable if medically managed and were restricted to conditions of hypertension and cholesterol and where biochemical data were within healthy ranges. History or current angina, transient ischemic attack, heart surgeries (stent insertion or other), or warfarin therapy were exclusions. CVD presence was categorically coded, and systolic/diastolic blood pressure assessed at orientation session for eligibility. Blood pressure data was collected via digital sphygmomanometer to obtain an average of three attempts and cross-checked against both radial and carotid pulse using SphygmoCor (AtCor Medical, Sydney, NSW, Australia) applanation tonometry, which incorporates participant demographics (gender, age, BMI) to assess normalcy of blood pressure. Cholesterol levels were collected from blood samples with results supplied by Australian Clinical Labs (Melbourne, VIC, Australia). On either of these measures, any levels deemed out of range (OOR) were excluded from the study and referred to their physician. Both blood pressure and cholesterol levels were assessed by the medical committee and participants enrolled if both were within normal ranges.

### 2.6. Medication Use

#### Cardiovascular Medication

Use of CVD medication was recorded, and included anti-hypertensives, statins, and maintenance antiplatelet medication including aspirin. Other medications were recorded into groups, e.g., non-steroidal anti-inflammatories (NSAIDs).

### 2.7. Supplements and Vitamins

Selected supplements were categorically recorded and grouped per category, (e.g., calcium and vitamin D; antioxidants; fish oil; multivitamins without B group). B-group vitamin use, and supplements geared to cognitive enhancement (e.g., *Gingko biloba*, *Bacopa monnieri*, ginseng) required wash-out of at least 4 weeks prior to enrolment into the study.

### 2.8. Body Mass Index

Body Mass Index score (BMI) [92] is a putative measure of body adiposity and signifying general health, and is the main measure used in international obesity guidelines including the World Health Organisation [55]. Body Mass Index is calculated from body weight (in kilograms) divided by height squared (in centimetres), to achieve a BMI score. Scores were also ordinally separated into four categories: underweight (<18.5), normal (18.5–24.9), overweight (25–29.9), and obese (>30).

### 2.9. Mood Outcomes

#### 2.9.1. Beck Depression Inventory-II (BDI-II) 

Depression symptoms were quantified via the BDI-II [93] Likert scale self-report inventory, widely-used in both clinical and research settings. The BDI-II has strong psychometric properties as an assessment tool among healthy older adults [94,95]. Higher scores on items such as sadness (e.g., ‘I feel sad much of the time’), indicated more severe depressive symptoms with a maximum score of 63. For the purposes of this study, scores were classified as follows: 1–10 (normal); 11–16 (mild mood disturbance); 17–20 (borderline clinical depression); 21–30 (moderate clinical depression); 31 and above (strong clinical depression).

#### 2.9.2. Chalder Fatigue Scale (CFQ) 

Participants completed the Chalder Fatigue Scale (14-item version, [96]), a self-report questionnaire for the measurement of mental and physical fatigue. The scale includes questions on cognitive function, memory, motivation, energy, interest in activities, and vigor, where higher scores indicate negative impacts on these factors. Reduction to motivation, energy levels, and cognitive abilities are typically observed as depression symptoms. The CFQ has been shown to be a valid measure of fatigue levels, with good internal consistency in non-clinical adults [97]. There are several ways to score the CFQ sanctioned by the authors, and for the purposes of this study the sum was derived Likert-style as 0,1,2,3 with a total score range 0–42.

#### 2.9.3. General Health Questionnaire (GHQ-12) 

The GHQ-12 [98] is a self-reporting measure of mental health, designed to assess recent change in function as well as appearance of psychiatric symptoms including psychological distress. The GHQ shows good validity in older samples [99]. As with the CFQ, there are several ways to score, and for this study the sum was derived Likert-style as 0–3 with a score range 0–36, with higher scores indicating greater negative impacts to self-rated general health.

### 2.10. Biochemical Measure: F_2_-Isoprostanes

**Figure A1:**
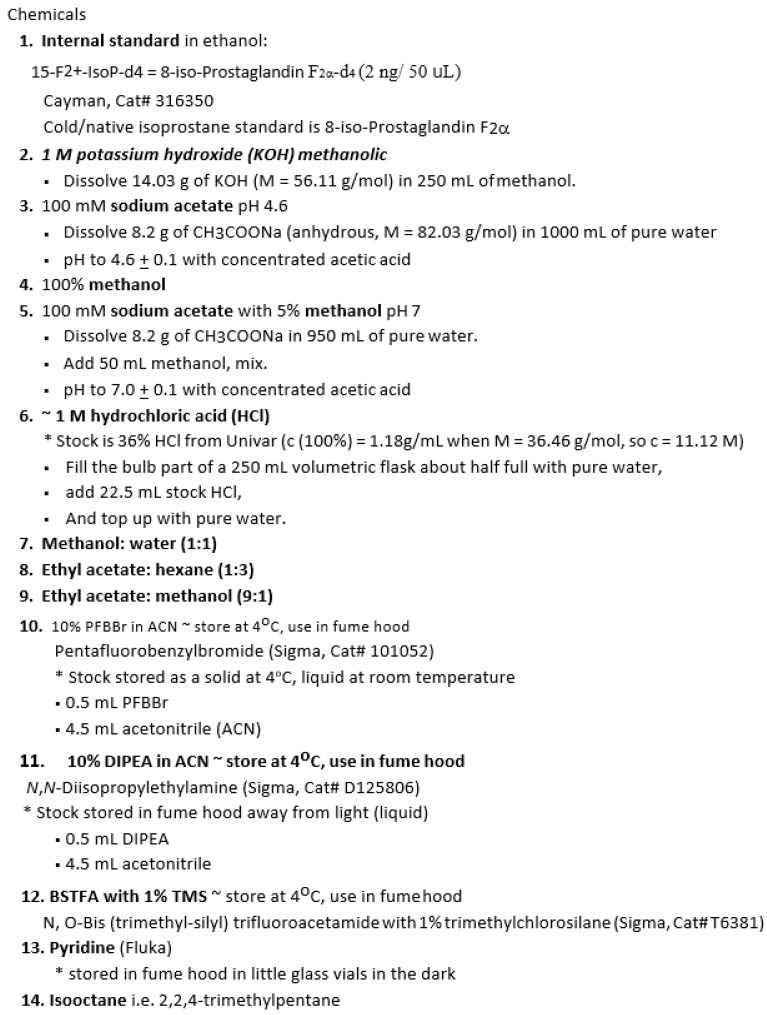
F_2_-Isoprostanes quantification processes.

**Figure A2:**
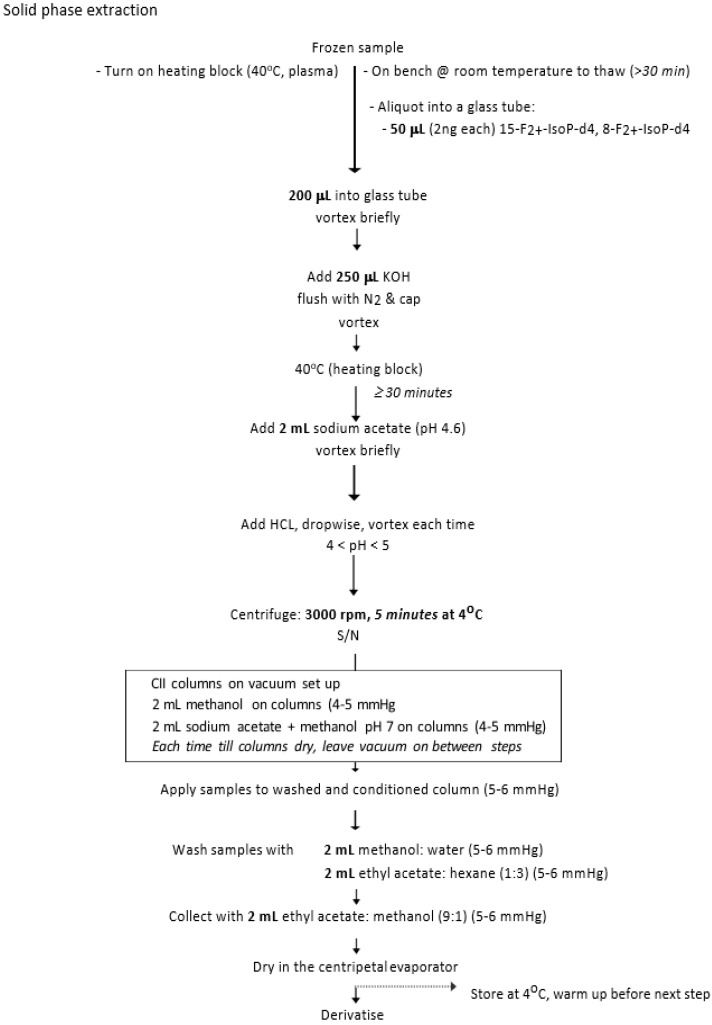
Solid Phase Extraction.

**Figure A3:**
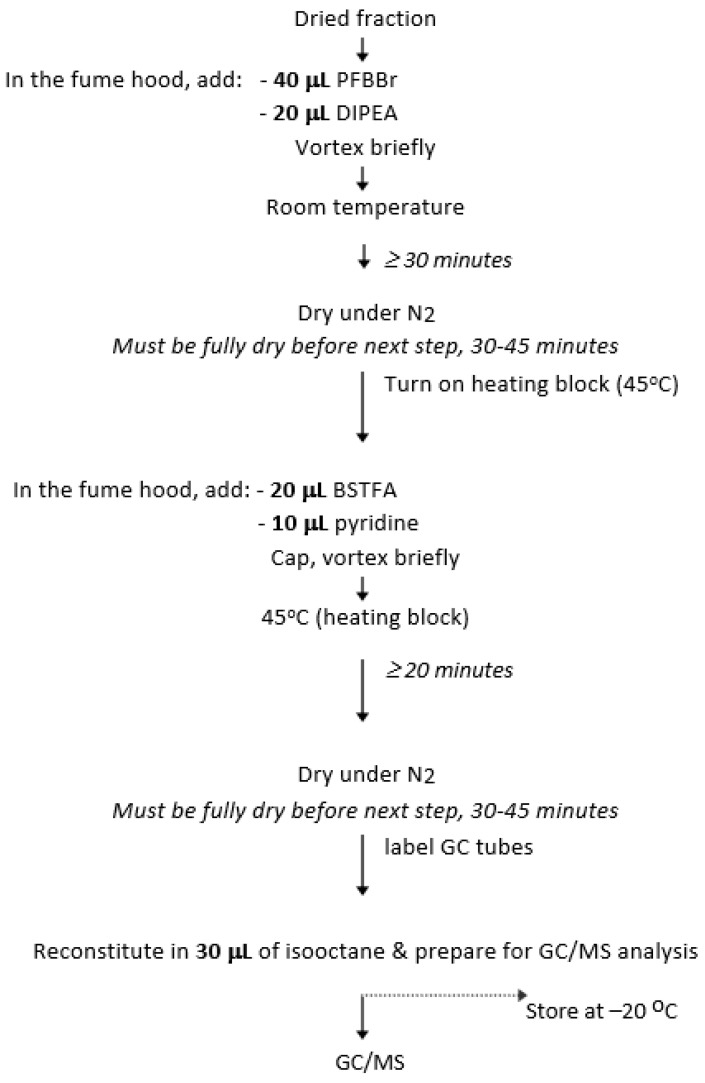
Derivatisation.

F_2_-Isoprostanes are prostaglandin-like molecules, derived from the non-enzymatic peroxidation of arachidonic acid [100]. They are detectable in a variety of tissues and fluids including plasma, urine, and cerebrospinal fluid and are considered a stable and consistent gold-standard quantification of OS [30]. Evidence indicates raised levels in many systemic pathologies [38,101,102] and levels are amenable to lifestyle and medication interventions [103,104]. F_2_-Isoprostanes were used in the current study as a measure of OS derived from plasma concentrations. Blood samples were collected from the cubital fossa region of each participant into a 10 mL EDTA vial before centrifuging at 3000× *g* for 10 min at 4 °C. To protect the samples from oxidation, 10 μL of butylated hydroxytoluene was added to vials prior to storage at −80 °C at Swinburne University of Technology. Frozen samples were transported to the University of Western Australia for assay (quantification procedure provided in Appendix A, Figure A1, Figure A2 and Figure A3). Values were reported in picomoles per litre (pmol/L) and log transformed to maintain standard distribution (refer Appendix B, Table A1). To assess post hoc group main effects differences, F_2_-Isoprostane levels were divided into four group ranges derived from calculated quartiles of the sample (25% incremental; refer to Table 1). Sex differences in F_2_-Isoprostane levels have been noted in several studies consistently reporting higher levels in females [63,85,105,106], so a sex split was utilised in quartiles calculations.

F_2_-Isoprostane quartile ranges were as follows:

### 2.11. Procedure

Participants attended the study site for two sessions within a 7-day period, for an initial screening visit and subsequent visit for data collection. Participants provided written and informed consent, prior to being screened for depressive symptoms and cognitive functioning with the GDS and MMSE, respectively. Eligible participants provided demographic information including age, sex, BMI, and years of education. Participant blood samples were collected at the second session following overnight fasting and without alcohol or caffeine consumption in the 24 h period prior.

### 2.12. Statistical Analysis

Data analyses were conducted using SPSS software, version 25.0 (IBM Corp., Armonk, NY, USA) [107]. Variables are presented as percentages, means, and standard deviation (±SD) for continuous measures, tallies and percentages for categorical variables. Relationships between screening and mood variables were examined using Pearson correlations. A General Linear Model (GLM) via factorial analysis of variance (ANOVA) was used with each mood measure as primary outcome variables and OS via F_2_-Isoprostane quartile groups, age, BMI as predictors. Differences were considered significant if *p*-value < 0.05 (two-sided).

All models were explored cross-sectionally, using baseline data, and therefore conclusions about changes in OS and mood over time cannot be inferred from this study.

## 3. Results

### 3.1. Demographics

Sociodemographic and clinical characteristics of the 568 participants are shown in Table 2. Overall participants’ mean age was 66.07 years (±4.09). Sex differences in several measures were also observed. MMSE data indicated a half mean point higher difference in female participants (*p* = 0.000). Male blood pressure scores were significantly higher, and they had a higher proportion of hypertension. Similarly, hypertensive medication was higher in males, and females reported higher corticosteroids/estrogens and mineral and vitamin D supplements. BMI weight ranges indicated that the proportion of female participants in the normal weight range was higher than that of males (41% and 27% respectively), conversely, more males were proportionately more likely to be overweight than females (52% and 36% respectively). No male in the study were reported as underweight. Pearson correlations between psychiatric variables demonstrated small to large positive and significant correlations (all *p* < 0.000) as shown in Table 3.

*T*-test analyses identified significant sex differences in F_2_-Isoprostane levels, *t*(411)–2.21, *p* = 0.028, where females were higher (M 935.57, SD 329.03) than males (M 856.77, SD 389.84), 95% C.I. [−148.94, −8.64]; subsequently, results are reported as a group but also separately for males and females to better explain the relationship between OS and mood.

### 3.2. Main Mood Outcomes

#### 3.2.1. Beck Depression Inventory-II

Depression incidence and severity in the current sample indicated that 95.1% of the sample had normal depression symptom levels. Mild depression symptoms were observed in 3.7% (*n* = 21) participants, and borderline clinical in 1.1% (*n* = 6), with one participant (0.2%), exhibiting BDI-II scores within the moderate clinical depression range.

**Figure 1:**
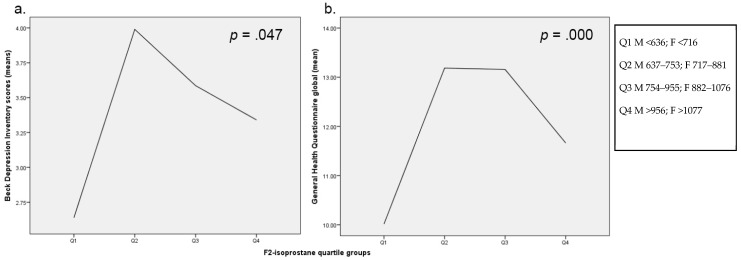
Estimated marginal means for F_2_-Isoprostane quartiles split for sex for (**a**) Beck Depression Inventory II, (**b**) General Health Questionnaire Scale.

Using the model for depression scores controlling for age, BMI, and sex, F_2_-Isoprostane quartile levels (refer Table 4 for multivariate models) were significantly associated with mood via BDI-II scores, F(3, 486) = 2.66, *p* = 0.047. BDI-II scores exhibited an inverted U-shape trend, where scores were highest in groups 2 and 3, and lowest in Group 1 of the F_2_-Isoprostane quartile groups, followed by group 4. Figure 1a illustrates mean BDI-II scores for each F_2_-Isoprostane group.

#### 3.2.2. Chalder Fatigue Scale

**Figure 2:**
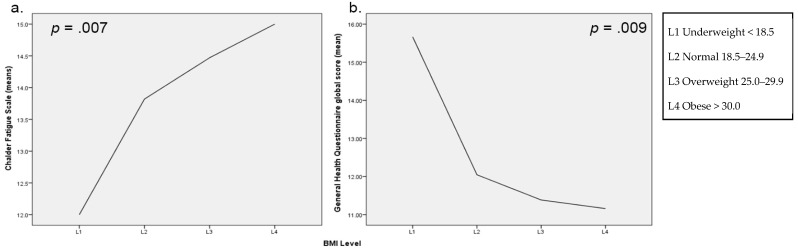
Means for BMI levels for (**a**) Chalder Fatigue Scale, and (**b**) General Health Questionnaire Scale.

In the model, BMI was significantly associated with CFS scores, F(6, 463) = 7.34, *p* = 0.007, 95% C.I. [0.02, 0.15], with these scores increasing with each 0.09 unit change of calculated BMI score. Figure 2a illustrates the mean Chalder Fatigue level across the BMI groups, indicating increases to self-rated fatigue with BMI levels.

#### 3.2.3. General Health Questionnaire

The GHQ scores were significantly associated with both F_2_ level—F(6, 474) = 10.25, *p* = 0.000, 95% C.I. [−3.38, −0.46]—and BMI, F(6, 474) = 6.98, *p* = 0.009, 95% C.I. [−0.28, −0.04]—in the model. F_2_-Isoprostane groups held an inverted-U shape trend on GHQ scores, with group 1 exhibiting the lowest GHQ scores followed by group 4, and groups 2 and 3 the highest, with a 1.44- and 1.38-point change to GHQ scores with increases in F_2_-Isoprostane scores, respectively (Figure 1b). BMI score held a negative relationship whereby GHQ scores decreased by 0.61 with increases to BMI (Figure 2b).

## 4. Discussion

The major finding in the current study is that OS levels predicted negative mood, whereby lower F_2_-Isoprostanes showed low BDI scores as well as better rated general health via the GHQ. Both measures showed increases to negative mood or problematic general health in quartiles 3 and 4 of F_2_-Isoprostanes, before dropping again with the fourth quartile of F_2_-Isoprostanes. A closer examination of the relationship between F_2_-Isoprostane levels and mood trends revealed some novel findings. The effect found of F_2_ quartiles on mood was non-linear, with a sine-shaped trend observed in mood between each F_2_-Isoprostane level Q1 and Q3 (lowest and medium), relative to Q4, were much lower, and Q2 (normative range) was most like Q4 (highest) in both of these outcome measures. One possible explanation is that higher F_2_-Isoprostane levels are reflective of a more elevated OS state occurring from what would ordinarily be Q1 or Q2 levels, meaning scores in the Q3/Q4 ranges reflect either acute variances of homeostatic state normally sitting at lower levels. In turn, elevated levels may produce the observed increased mood score, reflective of reduced positive mood. Given these participants were in good general health, it is feasible this relationship reflects an acute stress state from a challenge as a form of system response. It is also feasible that variances are a product of conditions associated with fluctuating F_2_-Isoprostane levels not accounted for in the data, such as antioxidant components of the diet, or presence of digestive system or respiratory conditions [40]. The use of quartile ranges exposed this trend that may not have been observed with scores alone.

These significant findings were partly consistent with previous meta-analyses which reported associations with depressive symptoms and increased OS in clinical and community adult samples [22,43,45]. However, the findings are inconsistent with a subsequent large-scale study (*n* = 2841) of adults aged 18–65 years from Black et al. [108], which found no significant associations between OS and depression in males or females. Notably, this study examined two measures of OS (plasma levels of F_2_-Isoprostanes, and 8-hydroxydeoxyguanosine); and the median age of participants was considerably younger (*m* = 42 years) than our sample (*m* = 65 years). Taken together, older adults may exhibit raised OS levels with increased negative mood in a similar pattern to clinical depression, reflecting common chronic and/or long-term systemic impacts. However, the exact mechanisms are not yet determined with this relationship, but consideration should be given to the fact that these levels of F_2_-Isoprostanes are still within healthy ranges. What is of clinical interest is that in a healthy older adult sample of strict inclusion criteria, with tightly controlled CVD and disease and lifestyle status, can still exhibit patterns between depression symptoms and OS observed in clinical groups. Subsequently, a link between a gold-standard measure of OS to mental health shows that biological mechanisms relate to modulation of mood in older adults.

There were significantly higher F_2_-Isoprostane levels found in females compared to males, suggesting that sex influences levels of OS. Although these findings are consistent with previous studies reporting sex differences in OS levels in both clinical and non-clinical populations [51,85,105,108,109], in these studies female F_2_-Isoprostane levels were typically lower than males. Previous research has suggested OS-related sex differences may be facilitated by the antioxidant capacity of oestrogen [109,110], which is markedly reduced post-menopause and equitable to levels seen in males [111], but taken with current findings, the relationship appears to be equivocal [111,112]. Although the current study did not collect estrogen level data to determine differences within non-clinical females, the data do suggest that estrogen levels could exert an influence on OS processes. Current study findings support sex differences in OS processes that should inform understanding of OS mechanisms occurring in older adults. However, as a corollary to these findings, research into F_2_-Isoprostanes differences in otherwise healthy cohorts is lacking in the literature, having only recently been examined more broadly [113], particularly with age as a factor, and represents an area of recommended further research.

In the prediction of mood in the models, sex-based differences were not observed as a significant predictor which suggests that the observable health differences above do not translate to sex-based biological influences on mood levels in this sample, despite the literature supporting a greater prevalence of depression in females aged over 60 years [38,39].

In this study the relationship of BMI to mood measures found a negative association with the GHQ (better self-rated mood with higher BMI), yet no relationship to depression symptoms via the BDI-II using the current models.

The Chalder Fatigue Scale results support the consensus that increased weight requires an increased energy expenditure in maintaining general function, thereby contributing to fatigue, but also that healthier weight may be facilitated by increased movement, which can improve energy levels. Both factors may serve to modify self-reported fatigue. This trend between self-rated energy levels and BMI observed in healthy older adults is concerning for the broader aging population in countries such as Australia, who in 2017–2018 had a higher proportion (78.2%) of older adults (65–74 years) considered overweight or obese [114]. Conversely, with the General Health Questionnaire, scores were negatively associated with BMI where lower scores imply better self-reported wellbeing. This pattern is also partly supported in the literature in non-clinical groups, which suggests that older cohorts in the overweight range have better mental health-related quality of life compared with other weight ranges, as well as younger comparators [115,116]. BMI is considered a modifiable risk factor, and not without contention in the literature as to its validity in older adults for long term cardiometabolic risk [60], or its use as an appropriate measure in older adults who may be low in BMI for reasons of muscle and bone density loss [117]. The findings here on BMI and mood level gives insight into self-perceived general health in older adults who are defined as overweight or obese according to BMI scores but who are otherwise in good health, without psychological disorders or symptoms.

There are some limitations of this study that may affect interpretation of the findings. Cross-sectional data cannot prove causality between mood and OS, age, sex, and BMI. Secondly, the sample comprised a restricted group (in terms of disease presence) of healthy older adults who may not be generally representative of the adult population aged 60–75 years. Female participants had significantly healthier BMI scores than males in the study, as well as BMI scores below the broader population for this age range [114]. As such, conclusions on the relationship between weight maintenance and mood in females are limited in broader application. Similarly, CVD presence (hypertension or hypercholesterolemia only) was medically managed with the need for cholesterol and blood pressure within normal ranges, but this presence may have produced impacts on other measures not accounted for. Finally, the study did not make comparisons to a clinical cohort possessing systemic conditions or psychiatric disorders, as the focus of the study was to understand the relationship between OS and mood in the otherwise healthy due to aging processes, with minimal confounds.

Although outside the scope of the current study, examination of dietary or supplement antioxidant use on the OS and mood relationship is also of interest, such as carotenoids, vitamin E, vitamin D, calcium, and minerals such as iron, zinc, and magnesium [118]. All play a role in maintaining health factors that influence oxidative stress status as we age, such as via homocysteine and glutathione levels [118,119]. Studies on the relationship between mood and antioxidant supplementation in older age groups are few but are generally positive and suggest antioxidant dietary supplementation may have a protective role in age-related cardiovascular and immune system conditions amenable to oxidative stress [120,121].

## 5. Conclusions

The current study found that elevated F_2_-Isoprostanes was associated with self-rated depressed mood and reductions to self-rated general health, suggesting a contribution of oxidative stress processes to maintenance of healthy mood. Higher BMI was associated with higher self-rated mood and general health, but increased Chalder Fatigue scores, which supports the indicator of a healthy sample with good quality of life but reinforcing links between healthy weight and energy levels.

Females had significantly higher F_2_-Isoprostanes, establishing the need to consider oxidative stress mechanisms in systemic pathologies for each sex. Future research examining the maintenance of healthy mood in older adults would benefit from integrating these sex differences, and BMI levels, hormones (corticosteroid or estrogens), and dietary or supplement use of antioxidants. Given the globally-aging population, a clearer understanding of how interrelated biological factors, in particular oxidative stress, contribute to the maintenance of mood is vital to quality of life as we age.

## Figures and Tables

**Table 1:** F_2_-Isoprostanes quartile calculations.

F_2_-Isoprostanes		
Quartiles	Females	Males
1	<716	<636
2	717–881	637–753
3	882–1076	754–955
4	>1077	>956

**Table 2:** Socio-demographic and clinical characteristics of participants.

Measures	Group	Males	Females	*t*-Value or χ^2^	*df*	Sex Difference *p*-Value *
Participants	568	216 (38%)	352 (61.9%)			
Age (years)	66.07 ± 4.09	66.30 ± 4.05	65.93 ± 4.12	1.02	566	0.308
Education (years)	16.20 ± 3.86	16.43 ± 3.99	16.03 ± 3.75	1.21	562	0.228
Body Mass Index (BMI)	26.47 ±4.74	26.78 ± 3.81	26.29 ± 5.23	1.19	549	0.234
Underweight (<18.5)	4 (0.7%)	0	4 (1.1%)	-	-	-
Normal (18.5–24.9)	195 (34.3%)	56 (25.9%)	139 (39.5%)	-	-	-
Overweight (25.0–29.9)	239 (42.0%)	112 (51.9%)	137 (36.1%)	-	-	-
Obese (>30.0)	113 (19.9%)	42 (19.4%)	71 (20.2%)	-	-	-
Screening measures						
Mini Mental State Examination score	28.82 ± 1.20	28.54 ± 1.33	28.99 ± 1.08	−4.35	533	0.000 *
Geriatric Depression Score (GDS)	3.15 ± 3.05	3.16 ± 3.05	3.15 ± 3.07	0.04	552	0.967
Cardiovascular features						
Cardiovascular condition	199 (27.3%)	77 (35.6%)	122 (34.7%)	0.061	563	0.805
Hypertension	117 (16%)	54 (25%)	63 (17.9%)	4.16	563	0.041 *
Cholesterol	75 (10.3%)	30 (13.9%)	45 (12.8%)	0.145	562	0.703
Systolic blood pressure (mm Hg)	129.55 ± 16.67	132.01 ± 16.07	128.04 ± 16.87	2.63	506	0.009 *
Diastolic blood pressure (mm Hg)	75.07 ± 10.60	76.56 ± 10.72	74.15 ± 10.43	2.50	506	0.013 *
Medication use						
Cardiovascular medications overall	174 (23.8%)	67 (31%)	107 (30.4%)	0.020	562	0.889
Hypertensive medication	102 (14%)	49 (22.7%)	53 (15.1%)	5.24	562	0.022 *
Statins	74 (10.1%)	30 (13.9%)	44 (12.5%)	0.219	562	0.640
Medications other	271 (37.1%)	103 (47.7%)	168 (47.7%)	0.001	562	0.973
Analgesics (e.g., paracetamol)	59 (8.1%)	21 (9.7%)	38 (10.8%)	0.173	562	0.678
Corticosteroid or estrogens	60 (8.2%)	13 (5%)	47 (13.4%)	7.67	562	0.006 *
NSAIDs	45 (6.2%)	17 (7.9%)	28 (8.0%)	0.002	562	0.965
PPIs	46 (6.3%)	17 (7.9%)	29 (8.2%)	0.027	562	0.870
Supplement and vitamin use						
Supplements and vitamins general	336 (46%)	110 (50.9%)	225 (63.9%)	9.40	562	0.002 *
Omega 3	160 (21.9%)	55 (25.5%)	105 (29.8%)	1.30	562	0.254
Vitamin D/calcium	192 (26.3%)	48 (22.2%)	144 (40.9%)	21.15	562	0.000 *
Glucosamine	93 (12.7%)	36 (16.7%)	57 (16.2%)	0.019	562	0.891
Minerals (iron, zinc, magnesium)	117 (16%)	30 (13.9%)	87 (24.7%)	9.69	562	0.002 *
Vitamin E supplement (antioxidant)	31 (4.2%)	7 (3.2%)	24 (6.8%)	3.34	562	0.068
Oxidative stress						
F_2_-Isoprostanes	904.66 ± 355.76	856.77 ± 389.84	935.57 ± 329.03	−2.21	411	0.028 *
Mood measures						
Beck Depression Inventory-II (BDI-II)	3.22 ± 3.92	3.56 ± 4.06	3.15 ± 3.84	0.58	496	0.563
Chalder Fatigue Scale	14.30 ± 3.08	14.46 ± 3.10	14.22 ± 3.05	0.82	472	0.412
General Health Questionnaire	11.57 ± 6.02	11.83 ± 6.16	11.43 ± 5.94	0.71	483	0.479

* *p*-Values significant where < 0.05. *df*—degrees of freedom; NSAID—non-steroidal anti-inflammatories; mm Hg—millimetres of mercury; POMS—Profile of Mood States; PPI—proton pump inhibitors.

**Table 3:** Pearson correlations of mood and depression outcome measures.

	GDS *n* = 555		CFS *n* = 473		GHQ *n* = 484		BDI *n* = 495	
	*r*	*p*-Value	*r*	*p*-Value	*r*	*p*-Value	*r*	*p*-Value
GDS	1	-	-	-	-	-	-	-
CFS	0.261 *	0.000	1	-	-	-	-	-
GHQ	0.182 *	0.000	0.125 *	0.000	1	-	-	-
BDI	0.536 *	0.000	0.349 *	0.000	0.152 *	0.000	1	-

* Correlation is significant where *p*-value < 0.01 (2-tailed). BDI-II—Beck Depression Inventory-II; CFS—Chalder Fatigue Scale; GDS—Geriatric Depression Scale; GHQ—General Health Questionnaire; *r*—Pearson correlation.

**Table 4:** Factorial ANOVA statistics for prediction of BDI-II, CFQ, and GHQ-12 measures.

Predictor Variables	Coefficient	95% C.I.	*t*	*p*
BDI-II				
F_2_ quartile 1	−0.60	−1.55, 0.36	−1.22	0.222
F_2_ quartile 2	0.67	−0.43, 1.77	1.19	0.234
F_2_ quartile 3	0.32	−0.78, 1.42	0.58	0.564
F_2_ quartile 4	0 ^b^	-	-	-
Sex—male	0.09	−0.62, 0.81	0.26	0.793
Sex—female	0 ^b^	-	-	-
Age (years)	0.05	−0.04, 0.13	1.12	0.264
BMI	0.07	−0.01, 0.14	−1.22	0.093
CF Scale				
F_2_ quartile 1	−0.98	−0.09, 0.68	−0.25	0.805
F_2_ quartile 2	0.04	−0.84, 0.93	0.09	0.925
F_2_ quartile 3	0.03	−0.88, 0.95	0.07	0.940
F_2_ quartile 4	0 ^b^	-	-	-
Sex—male	0.20	−0.37, 0.77	0.069	0.489
Sex—female	0 ^b^	-	-	-
Age (years)	−0.03	−0.09, 0.04	−0.76	0.447
BMI	0.09	0.02, 0.15	2.71	0.007 *
GHQ-12				
F_2_ quartile 1	−1.92	−3.38, −0.46	−2.59	0.010 *
F_2_ quartile 2	1.44	−0.23, 3.10	1.70	0.090
F_2_ quartile 3	1.38	−0.29, 3.05	1.62	0.106
F_2_ quartile 4	0 ^b^	-	-	-
Sex—male	0.38	−0.70, 1.46	0.69	0.492
Sex—female	0 ^b^	-	-	-
Age (years)	0.04	−0.09, 0.17	0.57	0.566
BMI	−0.16	−0.28, −0.04	−2.64	0.009 *

* significant at *p* < 0.05. ^b^—set as baseline; BMI—Body Mass Index; C.I.—confidence interval; *t*—*t*-test statistic.

## Data Availability

Data is contained within the article.

## Appendix B

**Table A1:** Log Calculations of F_2_-Isoprostanes.

Calculation			
amount (Isoprostanes) = area 569	x 2ng	x 1000 μL	x 1000 × 1000
in pmol/L area 573	standard	200 μL sample	354.5
